# Ambient air pollution is associated with pediatric pneumonia: a time-stratified case–crossover study in an urban area

**DOI:** 10.1186/s12940-019-0520-4

**Published:** 2019-08-28

**Authors:** Chi-Yung Cheng, Shih-Yu Cheng, Chien-Chih Chen, Hsiu-Yung Pan, Kuan-Han Wu, Fu-Jen Cheng

**Affiliations:** 1 Department of Emergency Medicine, Kaohsiung Chang Gung Memorial Hospital, No. 123, Dapi Rd., Niaosong Township, Kaohsiung, County 833 Taiwan; 2 Chang Gung University College of Medicine, No.259, Wenhua 1st Road, Guishan District, Taoyuan City, 333 Taiwan; 3 Department of Emergency Medicine, Yunlin Chang Gung Memorial Hospital, No. 1500, Gongye Rd, Mailiao Township, Yunlin County 638 Taiwan

**Keywords:** Particulate matter, Air pollution, Pediatric, Pneumonia, Season

## Abstract

**Background:**

Pneumonia, the leading reason underlying childhood deaths, may be triggered or exacerbated by air pollution. To date, only a few studies have examined the association of air pollution with emergency department (ED) visits for pediatric pneumonia, with inconsistent results. Therefore, we aimed to elucidate the impact of short-term exposure to particulate matter (PM) and other air pollutants on the incidence of ED visits for pediatric pneumonia.

**Methods:**

PM_2.5_, PM_10_, and other air pollutant levels were measured at 11 air quality-monitoring stations in Kaohsiung City, Taiwan, between 2008 and 2014. Further, we extracted the medical records of non-trauma patients aged ≤17 years and who had visited an ED with the principal diagnosis of pneumonia. A time-stratified case–crossover study design was employed to determine the hazard effect of air pollution in a total of 4024 patients.

**Results:**

The single-pollutant model suggested that per interquartile range increment in PM_2.5_, PM_10_, nitrogen dioxide (NO_2_), and sulfur dioxide (SO_2_) on 3 days before the event increased the odds of pediatric pneumonia by 14.0% [95% confidence interval (CI), 5.1–23.8%], 10.9% (95% CI, 2.4–20.0%), 14.1% (95% CI, 5.0–24.1%), and 4.5% (95% CI, 0.8–8.4%), respectively. In two-pollutant models, PM_2.5_ and NO_2_ were significant after adjusting for PM_10_ and SO_2_. Subgroup analyses showed that older children (aged ≥4 years) were more susceptible to PM_2.5_ (interaction *p* = 0.024) and children were more susceptible to NO_2_ during warm days (≥26.5 °C, interaction *p* = 0.011).

**Conclusions:**

Short-term exposure to PM_2.5_ and NO_2_ possibly plays an important role in pediatric pneumonia in Kaohsiung, Taiwan. Older children are more susceptible to PM_2.5_, and all children are more susceptible to NO_2_ during warm days.

## Introduction

Many epidemiological studies have reported that short-term variations in ambient air pollution are related to poor health outcomes, such as respiratory diseases, cardiovascular diseases, and mortality [1–4]. Particulate matter (PM), nitrogen dioxide (NO_2_), and ozone (O_3_) are reportedly related to hospital admissions for pneumonia [5]. Epidemiological studies suggest that fine particles (which are usually defined as PM having an aerodynamic diameter of < 2.5 μm; PM_2.5_) are more toxic than larger particles [6].

Pneumonia, an inflammatory lung condition, is the leading cause of death in children, accounting for approximately 1.3 million deaths among children aged < 5 years in 2010–2011 [7]. Air pollutants, such as PM_10_ (PM having an aerodynamic diameter of < 10 μm), PM_2.5_, NO_2_, and O_3_ are related to lung and systemic inflammation [8–10]. The health effects of air pollutants seemed to have regional and seasonal variations. The regional heterogeneity between the estimated effect of PM on hospitalization and mortality has been reported in several previous multi-city studies [1, 11]. These seasonal and regional variations might be explained by certain community characteristics, for instance, air conditioning [12], population density [13], the proportion of elderly residents [1], and effect modification by ambient temperature [2]. Previous studies also demonstrated that the effect of PM_2.5_ on emergency hospitalizations for pneumonia [14] was greater for children and its effect on out-of-hospital cardiac arrest (OHCA) was greater in elderly patients [15]. For children, air pollution was found to be associated with emergency department (ED) admission for respiratory diseases and asthma [16, 17]. However, only a limited number of studies have focused on air pollution and ED visits for pediatric pneumonia, and the results are inconsistent [18, 19]. Furthermore, only limited information is available regarding the pediatric populations which are particularly susceptible to these exposures.

Over a 7-year period, in South Taiwan, we collected data of pediatric patients who presented at the ED from a tertiary academic medical center due to pneumonia. Using a case–crossover design, the data were analyzed with respect to weather and air pollution parameters. The study aimed: (1) to evaluate correlation between increase in short-term exposure to air pollutants and events of pediatric pneumonia and (2) to evaluate the potential triggering effects of PM_2.5_, especially in individuals with pre-existing disease.

## Materials and methods

### Kaohsiung City

Kaohsiung is located in the southwestern part of Taiwan and has a tropical monsoon climate. It is the leading industrial city with the largest commercial harbor in Taiwan. The industry clusters include basic metals, nonmetallic mineral products, transportation equipment manufacturing, food and agricultural products, chemical products, machinery and repairs, and power equipment and repairs.

### Study population

This was a retrospective observational study conducted in an urban tertiary medical center, which has 72,000 ED visits on an average every year. The study period was from January 1, 2008 to December 31, 2014. We retrospectively reviewed the electronic medical records, and from the ED’s administrative database, extracted data of non-trauma patients who were ≤ 17 years old and had visited an ED with a documented pneumonia diagnosis (International Classification of Diseases, ninth revision [ICD-9]: 480–486). We abstracted the following characteristics from the electronic charts: age, sex, and underlying conditions, including respiratory diseases (such as chronic respiratory failure and restrictive lung), cerebral palsy, asthma, and epilepsy.

This study was approved by the institutional review board of our hospital (no. 201801301B0) and has been performed in accordance with the ethical standards of the 1964 Declaration of Helsinki and its later amendments. For this type of study, informed consent from the subjects was not required.

### Pollutant and meteorological data

In 1994, Taiwanese Environmental Protection Administration, a government agency, had constructed 11 air quality monitoring stations in Kaohsiung City. The commercial monitoring instruments of the stations were designated by the US Environmental Protection Agency as equivalent or reference instruments and manufactured by US Thermo Environmental Instruments, Inc. (Franklin, MA, USA). The automatic stations routinely monitor several “criteria” pollutant levels, including NO_2_ (using ultraviolet fluorescence), sulfur dioxide (SO_2_, using ultraviolet fluorescence), PM_10_ (using beta-ray absorption), PM_2.5_ (using beta-ray absorption), and O_3_ (using ultraviolet photometry), as well as weather condition, such as temperature and humidity. Missing data accounted for less than 1% of the total data.

From all the monitoring stations, we collected air pollution data on an hourly basis, and collected addresses of pediatric pneumonia patients from medical records. In addition, we collected recordings of mean temperature and humidity on a daily basis. Finally, from the nearest monitoring station, we computed the 24-h average pollutant levels.

### Statistical analysis

To analyze pediatric pneumonia events, we employed a time-stratified case–crossover study design [20, 21] as an alternative to the Poisson time series regression models for estimating acute episodic events following short-term exposure attributed to air pollutants. We investigated single-day lags from the current day (lag 0) and each of 1–3 days before the pediatric pneumonia event (lag 1, lag 2, and lag 3). We performed within-subject comparisons between case and control periods. The date of the pediatric pneumonia event was defined as case period. Time was stratified into separate months to select control periods as the days falling on the same day of the week in the same month of the same year as the case period. This self-matching control period selection strategy was considered to adjust for the effects of long-term trends, seasonality, and day of the week [22]. Using conditional logistic regression, the odds ratios (ORs) and 95% confidence intervals (CIs) of the pediatric pneumonia cases associated with PM_2.5_ mass and each air pollutant were estimated. Subgroup analyses including sex, age, and underlying diseases in the most susceptible groups were also performed. Exposure levels to air pollutants were included into the pollutant models as continuous variables. Each model was adjusted for meteorological variables, such as average daily temperature and humidity on the same day and during lag intervals. Our analysis was conducted in two steps. First, conditional logistic regression analysis was performed using the SPSS version 25.0 software. The baseline model included a linear expression that included air pollutants and confounding factors, such as temperature and humidity. Second, we examined nonlinear effects by introducing temperature and humidity separately in the model and comparing the goodness-of-fit using the Akaike information criterion (AIC). The second step was performed using the SAS macro lgtphcurv9 (in SAS version 9.4), which implements natural cubic spline methodology to fit a potentially nonlinear response curve in conditional logistical regression models for matched case–control studies. With temperature, the AIC value for the linear model (11,160.162) was better than that for the spline model (11,161.772), and the test of curvature (nonlinear relationship) was nonsignificant (*p* = 0.30). Similarly, with humidity, the AIC value for the linear model (11,146.628) was better than that for the spline model (11,149.575), and the test of curvature was nonsignificant (*p* = 0.59). As a result, we used the linear model for the entire conditional logistic regression analysis.

The ORs were calculated based on interquartile range (IQR) increments in PM_2.5_, PM_10_, NO_2_, SO_2_, and O_3_ exposure. The significance criterion was set at *p* < 0.05. All statistical analyses were performed with SPSS version 25.0 (IBM Corp, Armonk, NY, USA).

## Results

In total, 4625 pediatric pneumonia cases were recorded in Kaohsiung over the 7-year study period. Of these, 601 patients were excluded because they were not residents of Kaohsiung City; whereas the other 4024 patients were included in the study. Table 1 lists the demographic characteristics of the 4024 patients. Among them, 2144 (53.3%) patients were male and the mean age was 5.0 ± 3.6 years. In all, 305 (7.6%) cases had respiratory disease, 250 (6.2%) had asthma, 124 (3.1%) had cerebral palsy, and 110 (2.7%) had epilepsy. Of the total, 2192 (54.5%) cases occurred during the warm season (April to September), whereas 1762 (43.8%) occurred during warm days (≥26.5 °C).

**Table 1:** Characteristics of the cases (*n* = 4024)

Characteristic	Number	%
Age (mean ± SD)	5.0 ± 3.6	
Male sex	2144	53.3
Respiratory disease	305	7.6
Asthma	250	6.2
Cerebral palsy	124	3.1
Epilepsy	110	2.7
Warm season	2192	54.5
Warm days (≥26.5 °C)	1762	43.8

Table 2 lists the meteorological factors, daily mean concentrations of air pollutants and weather variables in Kaohsiung during the study period. The average PM_2.5_ and PM_10_ concentrations over the study period were 41.1 and 72.8 μg/m^3^, respectively. The average NO_2_, SO_2_, and O_3_ levels were 19.1, 6.4, and 29.1 ppb, respectively.

**Table 2:** Summarized statistics for meteorology and air pollution in Kaohsiung, 2008–2014

Percentiles							
	Minimum	25%	50%	75%	Maximum	Mean	IQR
PM_2.5_ (μg/m^3^)	3.6	23.2	40.5	54.6	126.7	41.1	31.4
PM_10_ (μg/m^3^)	14.7	43.1	70.7	95.7	582.0	72.8	52.6
NO_2_ (ppb)	3.9	13.2	18.4	24.3	24.3	19.1	11.1
SO_2_ (ppb)	1.8	4.9	6.1	7.8	17.2	6.4	2.9
O_3_ (ppb)	3.5	19.0	28.1	37.5	74.6	29.1	18.5
Temperature (°C)	12.4	22.3	26.4	28.8	32.1	25.3	6.5
Humidity (%)	44.0	70.0	74.0	78.1	95.3	74.0	8.1

The missing data for all monitor stations were less than 1%

Table 3 shows the Pearson’s correlation coefficients for the weather and air pollutant conditions. PM_2.5_ was highly correlated with PM_10_ (*r* = 0.915; *p* < 0.0001) and NO_2_ (*r* = 0.802, *p* < 0.0001); and moderately correlated with SO_2_ (*r* = 0.516, *p* < 0.0001) and O_3_ (*r* = 0.427, *p* < 0.0001).

**Table 3:** Spearman correlation coefficients between air pollutants and weather conditions during the 7-year study period (upper right triangle), and during the warm period (lower left triangle)

	PM_2.5_	PM_10_	NO_2_	SO_2_	O_3_	Temperature	Humidity
PM_2.5_	1.000	0.915	0.802	0.516	0.427	−0.570	−0.406
PM_10_	0.915	1.000	0.758	0.472	0.422	−0.544	−0.441
NO_2_	0.694	0.705	1.000	0.509	0.116	−0.758	−0.323
SO_2_	0.396	0.392	0.481	1.000	0.209	−0.206	−0.302
O_3_	0.775	0.750	0.489	0.226	1.000	0.068	−0.397
Temperature	−0.355	−0.393	−0.589	−0.163	−0.296	1.000	0.257
Humidity	−0.233	−0.266	−0.054	−0.120	−0.303	−0.258	1.000

**Fig. 1:**
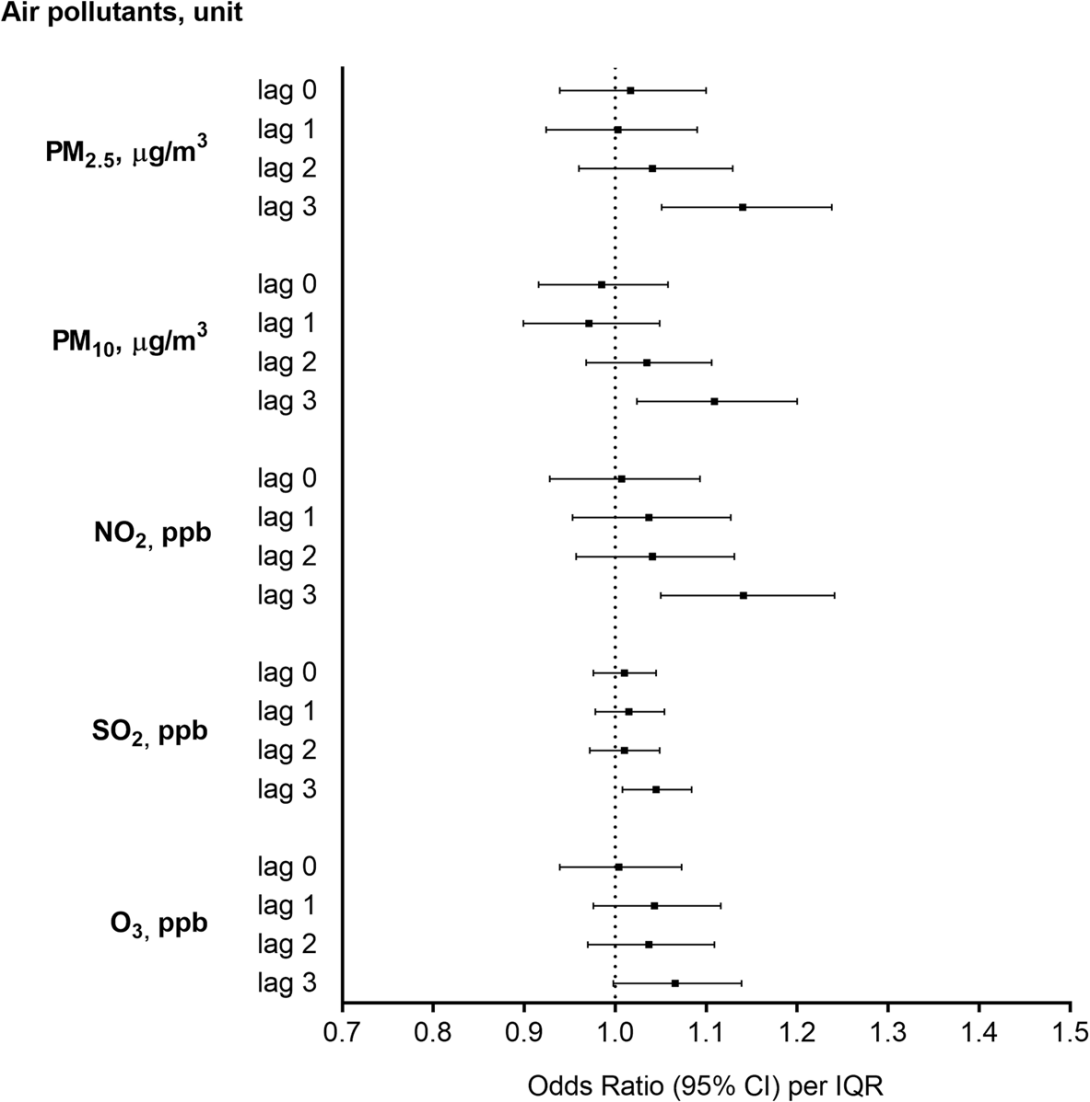
Odds ratios (ORs) and 95% confidence intervals (CIs) for pediatric pneumonia ED visits associated with IQR increments in air pollutant levels, with adjustment for temperature and humidity. ED, emergency department; IQR, interquartile range

Figure 1 shows the year-round estimates of the pollutants’ effects on pediatric pneumonia ED visits after adjustment for temperature and humidity. IQR increases in PM_2.5_, PM_10_, NO_2_, and SO_2_ levels on lag 3 were associated with increments of 14.0% (95% CI, 5.1–23.8%), 10.9% (95% CI, 2.4–20.0%), 14.1% (95% CI, 5.0–24.1%), and 4.5% (95% CI, 0.8–8.4%) in the odds of pediatric pneumonia ED visits, respectively. Meanwhile, the IQR increase in O_3_ level was associated with a 6.6% (95% CI, − 0.2–13.9%), not significantly related to pediatric pneumonia ED visits.

A two-pollutant model was used to determine which individual contaminant influences the number of pediatric pneumonia ED visits independently of other pollutant effects. In accordance with the results obtained from the single-pollutant models, the multi-pollutant models were fitted with different pollutant combinations (with up to two pollutants per model) to assess the stability of the effects of PM. The results obtained are presented in Table 4. An IQR increase in PM_2.5_ was significantly related to ED visits for pediatric pneumonia after adjustment for PM_10_ (OR = 1.179, 95% CI: 1.009–1.378%) and SO_2_ (OR = 1.119, 95% CI: 1.027–1.219%). An IQR increase in NO_2_ was significantly associated with ED visits for pediatric pneumonia after adjustment for PM_10_ (OR = 1.106, 95% CI: 1.004–1.218%) and SO_2_ (OR = 1.115, 95% CI: 1.015–1.226%). Associations with pneumonia ED visits were no longer significant when PM_2.5_ was adjusted for NO_2_ (OR = 1.089, 95% CI: 0.986–1.202%) or when NO_2_was adjusted for PM_2.5_ (OR = 1.088, 95% CI: 0.984–1.203%) in two-pollutant models.

**Table 4:** Emergency department visits for each interquartile range change in the two-pollutant models

OR (95% CI) of pneumonia Adjusted for temperature, humidity, and pollutant					
Single-pollutant model		Adjusted PM_2.5_	Adjusted PM_2.5–10_	Adjusted NO_2_	Adjusted SO_2_
PM_2.5_	1.140 (1.051–1.238)		1.156 (1.055–1.267)	1.089 (0.986–1.202)	1.119 (1.027–1.219)
NO_2_	1.141 (1.050–1.241)	1.088 (0.984–1.203)	1.136 (1.040–1.240)		1.115 (1.015–1.226)
SO_2_	1.045 (1.008–1.084)	1.030 (0.991–1.070)	1.042 (1.004–1.082)	1.022 (0.981–1.066)	

PM_2.5–10_: Particulate matter with an aerodynamic diameter between 2.5 and 10 μm.

**Fig. 2:**
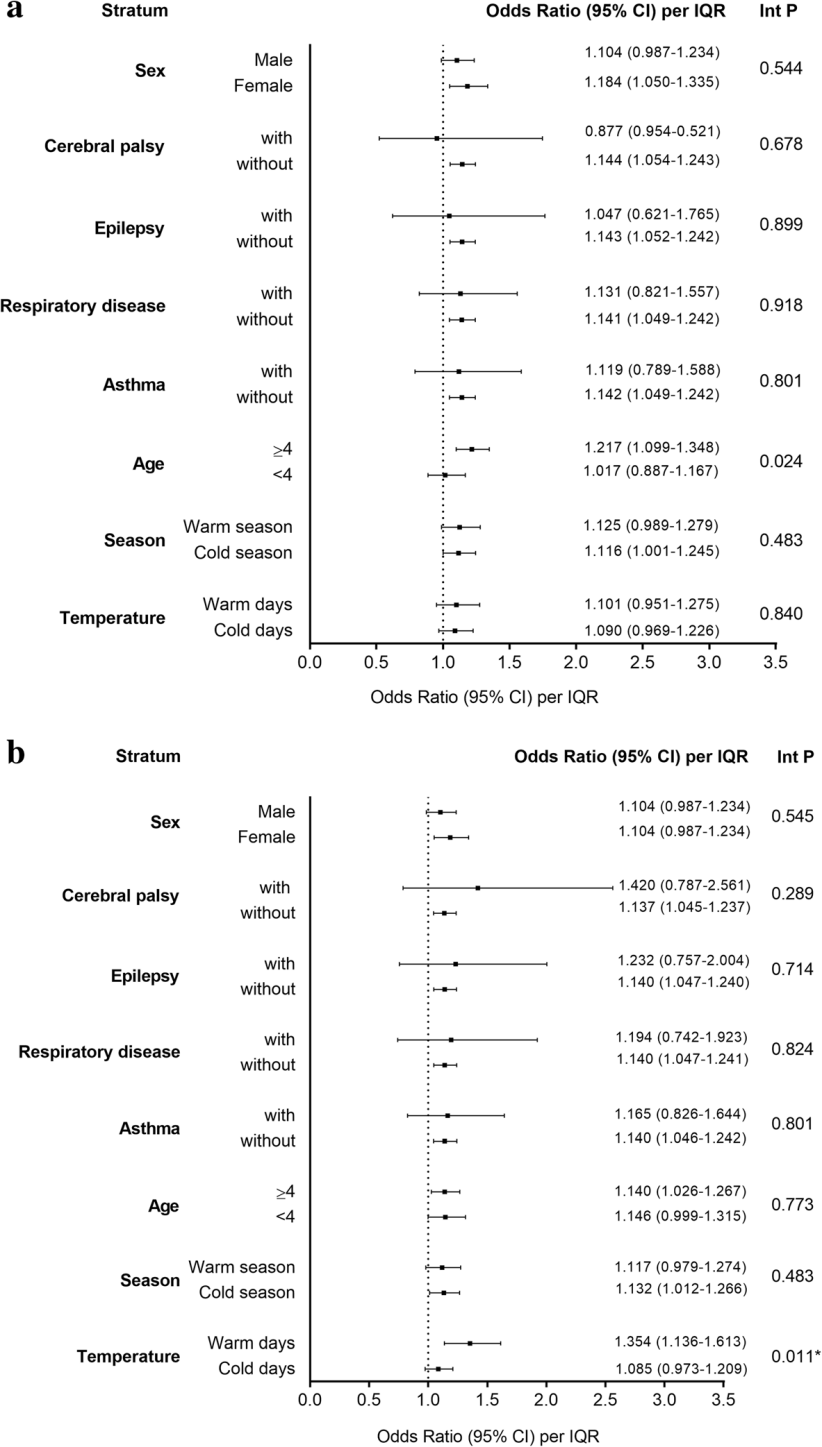
Odds ratios (ORs) for IQR increments in (**a**) PM_2.5_ and (**b**) NO_2_ on lag 3 after adjustment for temperature and humidity. The x-axis represents OR with 95% confidence intervals (CIs). The warm season was from April to September. *p < 0.05. Int P, interaction *p*-value; IQR, interquartile range

Figure 2 presents the results of the stratified analysis to examine the effect of PM_2.5_ and NO_2_ on pediatric pneumonia according to different seasons, temperature, and underlying diseases on lag 3, after adjustment for temperature and humidity. As shown in Fig. 2a, older children (aged ≥4 years) were more susceptible to PM_2.5_; an increase in the IQR for PM_2.5_ was associated with increases in the odds of ED visits for pneumonia of 21.7% (95% CI, 9.9–34.8%) for older children and 1.7% (95% CI, − 11.3– 16.7%; interaction *p* = 0.024) for younger children (aged < 4 years). The children were more susceptible to NO_2_ during the warm days (≥26.5 °C) than during the cool days (< 26.5 °C) (Fig. 2b), an increase in the IQR for NO_2_ was associated with increases of 35.4% (95% CI, 13.6–61.3% and 8.5% (95% CI, − 2.7– 20.9%; interaction *p* = 0.011) in the odds of ED visits for pneumonia, respectively. There were no significant differences with respect to the effects of PM_2.5_ or NO_2_ on pediatric pneumonia between the male and female patients, between those with or without underlying cerebral palsy, epilepsy, respiratory disease, or asthma, and between different seasons or temperatures.

## Discussion

In this study, we estimated the effects of PM and other air pollutants on pediatric pneumonia and found that PM_2.5_ and NO_2_ possibly play important roles in pediatric pneumonia events in Kaohsiung, Taiwan. Of all pollutant exposures included in the analysis, the odds of pediatric pneumonia following PM_2.5_ exposure was greater in older children. Additionally, the effect of NO_2_ on pediatric pneumonia was more significant during warm days.

Recently, many studies have focused on the association between PM_2.5_ and human health, especially in respiratory and cardiovascular diseases. Several previous studies demonstrated a positive association between PM_2.5_ and pediatric respiratory disease, such as upper respiratory infection and asthma [23, 24]. Some epidemiological studies have also demonstrated positive associations between PM_2.5_ and pediatric pneumonia. Lv et al. demonstrated increased risk of hospital admissions for pediatric pneumonia for PM_2.5_ on lag 4 [18]. Xiao et al. reported that air pollutants, including PM_2.5_, were associated with pediatric ED visits for respiratory tract infections on lag 0–3 [25]. On the other hand, some studies did not show statistically significant associations of the short-term effect of PM_2.5_ concentrations on pediatric pneumonia [24, 26, 27]. The difference between these studies should be considered with caution for several reasons. First, Strickland et al. examined lag 0–1 [24], and Malig et al. examined lag 0–2 [27], which revealed no statistically significant association between PM_2.5_ and pneumonia. Our study demonstrated a positive correlation between PM_2.5_ and pediatric pneumonia ED visits on lag 3; Lv et al.’s study on lag 4 and Xiao et al.’s study on lag 0–3 had the same result [18, 25]. Strickland et al.’s and Malig et al.’s studies did not examine the day before admission eariler than lag 1 and lag 2 [24, 27], respectively, and this difference may have contributed to the different results. Second, the different chemical components of PM_2.5_ may have different health effects. Darrow et al. observed that the carbon fraction of PM_2.5_, particularly organic carbon, was positively associated with pediatric pneumonia, but the total PM_2.5_ showed a negative association [26]. Xiao et al. estimated joint effects from O_3_ and PM_2.5_ components SO_4_^2−^, NO_3_^−^, and NH_4_^+^, and found the result was significantly associated with increased odds of pediatric pneumonia [25]. The different PM components of different regions may have contributed to different results.

PM_2.5_ has been reported to contribute to varying human health effects in different age groups. A case–crossover study found people of advanced age were more susceptible to the adverse effects of PM_2.5_ on OHCA [15]. Few studies have performed age group analyses of the effects of PM on pediatric pneumonia. Darrow et al. reported that hazard ratios tended to be higher in children aged 1–4 years compared with infants less than 1 year of age on pediatric pneumonia, but the interaction *p* values were not calculated in the study [26]. Lv et al. implied that young children (< 1 year) were at the highest risk of hospital admission for pneumonia due to airborne PM, but the interaction p values were also not evaluated [18]. The present study demonstrated that the odds of pediatric pneumonia following PM_2.5_ exposure was greater in older children (≥4 years old, interaction *p* = 0.024). One possible reason for this result was that children of different ages might spend different amounts of time outdoors, leading to varying air pollution exposure. Furthermore, air pollutants seem to have varying health effects on different age groups. Hassanvand et al. found that PM was not associated with increased high-sensitivity C-reactive protein (hsCRP) in healthy young adults, whereas in elderly subjects, hsCRP increased with PM_2.5_ exposure [8]. Using urinary malondialdehyde as a biomarker of oxidative stress, Kim et al. concluded that elderly adults are more susceptible than young children to ambient fine particulates and related oxidative stress [28].

Several studies tried to identify the mechanism by which PM contributes to pneumonia. An animal study found that PM_2.5_ exposure was associated with increased levels of DNA lesions in mouse lungs [29]. Through microfluidic chips, Schulze et al. concluded that PM_2.5_ interferes with alveolar macrophages, and pulmonary epithelial cells stimulate the release of a variety of cytokines and lead to inflammation [30]. Hassanvand et al. provided evidence that short-term exposure to PM_2.5_ was linked to elevated inflammation and coagulation of blood markers [8]. Zhang et al. reported that short-term PM_2.5_ exposure was associated with airway inflammation in school children [31]. A review article demonstrated that PM_2.5_ and PM_10_ exposure was associated with increased instances of pediatric pneumonia [19]. The present study also supported these results, and we found a positive association between PM_2.5_ and pediatric pneumonia ED visits.

NO_2_ has been found to be associated with all-cause mortality [32], cardiovascular mortality [33], hospital admissions for cardiovascular disease [34], and admission for pneumonia [35]. Toxicological studies have suggested that NO_2_ might damage macrophages, natural killer cells, and CD4 to CD8 ratios in the respiratory tract, leading to decreased mucociliary clearance and vulnerable respiratory epithelium [36]. However, the association between NO_2_ and pediatric pneumonia remains controversial. Some studies demonstrated the acute effect of NO_2_ exposure leading to ED visits for pediatric pneumonia [26, 37]. On the other hand, another study did not find a positive effect between NO_2_ and pediatric pneumonia [25]. A systematic review including 17 studies concluded that NO_2_was associated with an increase in hospital admissions due to pediatric pneumonia [19]. The current study had a similar result, and we found that NO_2_ might play an important role in pediatric pneumonia ED visits.

Seasonal variation seems to play an important role on air pollutants and human health. Szyszkowiczet al. demonstrated that the associations between air pollutants and respiratory health outcomes were stronger during the warm season [38]. Dong et al. implied the effects of NO_2_ and SO_2_ on daily ischemic stroke counts were stronger in the cold season than in the warm season [39]. Cheng et al. found a stronger association between PM_2.5_ and pneumonia with septicemia ED visits during the warm season [4]. Ueda et al. found that PM_2.5_ mass correlated with increased all-cause mortality, especially in transitional seasons rather than in summer and winter [40]. Few studies have focused on the seasonal effects of air pollution on pediatric pneumonia. Lv et al. revealed that children were at higher risk of hospital admission for pneumonia due to airborne PM, particularly on warm days [18]; but seasonal differences were not observed in another study [26]. In addition, although the risk was higher on warm days in Lv et al.’s study [18], the interaction *p*-value was not calculated in that study. The present study revealed that ORs for NO_2_were higher during the warm days (*p* = 0.011). One possible reason for this result is that variable concentrations of air pollutants and each PM component may relate to seasonal differences in human health [40, 41]. Seasonal variations may also result from different patterns of exposure to air pollutants. People tend to stay inside when weather conditions are extremely hot or cold, and thus decrease their exposure to outdoor air pollutants. When staying indoors, a positive correlation between the numbers of open windows for ventilation and exposure to air pollutants was observed [42]. In addition, meteorological factors might affect the health effect of air pollutants. Huang et al. demonstrated that the combination of low temperatures and high PM was associated with a greater incidence of developing acute coronary syndrome [43]. The combination of weather variation and lifestyle change might lead to the different health effects of air pollutants.

There are certain limitations to our study. First, the study was conducted in an industrial city having a tropical monsoon climate; the mixture of air pollutants and seasonal effects may be different in other regions. Second, as an exposure estimate for the entire population, we analyzed air pollution data from fixed monitoring sites assuming the exposure to be homogenous across the whole area. Factors such as personal protective equipment use and time spent outdoors may affect personal exposure. Third, individuals were identified in a single tertiary medical center, which limited the sample size. Moreover, individual susceptibility might vary due to ethnic differences. Thus, further studies should be conducted in more regions with larger samples and include seasonal constituent analysis.

## Conclusions

We found that PM_2.5_ and NO_2_ possibly play important roles in pediatric pneumonia events in Kaohsiung. Older children were found to be more susceptible to the adverse effects of PM_2.5_. Additionally, the health effects of the different air pollutants varied with temperature and patients were more susceptible to NO_2_ during the warm days.

## Data Availability

The datasets used and analyzed during the current study are available from the corresponding author on reasonable request.

## Abbreviations

CI: Confidence interval
ED: Emergency department
IQR: Interquartile range
NO_2_: Nitrogen dioxide
O_3_: Ozone
OHCA: Out-of-hospital cardiac arrest
OR: Odds ratios
PM: Particulate matter
PM_10_: Particulate matter with diameter < 10 μm
PM_2.5_: Particulate matter with diameter < 2.5 μm
SD: Standard deviation
SO_2_: Sulfur dioxide

## References

[1] Kim TY, Kim H, Yi SM, Cheong JP, Heo J (2018). Short-term effects of ambient PM2.5 and PM2.5-10 on mortality in major cities of Korea. Aerosol Air Qual Res.

[2] Cheng MH, Chiu HF, Yang CY (2015). Coarse particulate air pollution associated with increased risk of hospital admissions for respiratory diseases in a tropical city, Kaohsiung, Taiwan. Int J Environ Res Public Health.

[3] Weichenthal S, Kulka R, Lavigne E, Van Rijswijk D, Brauer M, Villeneuve PJ, Stieb D, Joseph L, Burnett RT (2017). Biomass burning as a source of ambient fine particulate air pollution and acute myocardial infarction. Epidemiology.

[4] Cheng FJ, Lee KH, Lee CW, Hsu PC (2019). Association between particulate matter air pollution and hospital emergency room visits for pneumonia with septicemia: a retrospective analysis. Aerosol Air Qual Res.

[5] Cheng MF, Tsai SS, Chiu HF, Sung FC, Wu TN, Yang CY (2009). Air pollution and hospital admissions for pneumonia: are there potentially sensitive groups?. Inhal Toxicol.

[6] Ren M, Fang X, Li M, Sun S, Pei L, Xu Q, Ye X, Cao Y (2017). Concentration-response relationship between PM2.5 and daily respiratory deaths in China: A systematic review and metaregression analysis of time-series studies. Biomed Res Int.

[7] Walker CLF, Rudan I, Liu L, Nair H, Theodoratou E, Bhutta ZA, O'Brien KL, Campbell H, Black RE (2013). Global burden of childhood pneumonia and diarrhoea. Lancet.

[8] Hassanvand MS, Naddafi K, Kashani H, Faridi S, Kunzli N, Nabizadeh R, Momeniha F, Gholampour A, Arhami M, Zare A, Pourpak Z (2017). Short-term effects of particle size fractions on circulating biomarkers of inflammation in a panel of elderly subjects and healthy young adults. Environ Pollut.

[9] Ji X, Han M, Yun Y, Li G, Sang N (2015). Acute nitrogen dioxide (NO_2_) exposure enhances airway inflammation via modulating Th1/Th2 differentiation and activating JAK-STAT pathway. Chemosphere.

[10] Xing YF, Xu YH, Shi MH, Lian YX (2016). The impact of PM2.5 on the human respiratory system. J Thorac Dis.

[11] Bell ML, Ebisu K, Peng RD, Walker J, Samet JM, Zeger SL, Dominici F (2008). Seasonal and regional short-term effects of fine particles on hospital admissions in 202 US counties, 1999-2005. Am J Epidemiol.

[12] Bell ML, Ebisu K, Peng RD, Dominici F (2009). Adverse health effects of particulate air pollution:modification by air conditioning. Epidemiology..

[13] Zeka A, Zanobetti A, Schwartz J (2005). Short term effects of particulate matter on cause specific mortality: effects of lags and modification by city characteristics. Occup Environ Med.

[14] Qiu H, Tian LW, Pun VC, Ho KF, Wong TW, Ignatius TS (2014). Coarse particulate matter associated with increased risk of emergency hospital admissions for pneumonia in Hong Kong. Thorax.

[15] Xia R, Zhou G, Zhu T, Li X, Wang G (2017). Ambient air pollution and out-of-hospital cardiac arrest in Beijing. China Int J Environ Res Public Health.

[16] Bono R, Romanazzi V, Bellisario V, Tassinari R, Trucco G, Urbino A, Cassardo C, Siniscalco C, Marchetti P, Marcon A (2016). Air pollution, aeroallergens and admissions to pediatric emergency room for respiratory reasons in Turin, northwestern Italy. BMC Public Health.

[17] Lim H, Kwon HJ, Lim JA, Choi JH, Ha M, Hwang SS, Choi WJ (2016). Short-term effect of fine particulate matter on children's hospital admissions and emergency department visits for asthma: a systematic review and meta-analysis. J Prev Med Public Health.

[18] Lv C, Wang X, Pang N, Wang L, Wang Y, Xu T, Zhang Y, Zhou T, Li W (2017). The impact of airborne particulate matter on pediatric hospital admissions for pneumonia among children in Jinan, China: a case-crossover study. J Air Waste Manage Assoc.

[19] Nhung NTT, Amini H, Schindler C, Joss MK, Dien TM, Probst-Hensch N, Perez L, Künzli N (2017). Short-term association between ambient air pollution and pneumonia in children: a systematic review and meta-analysis of time-series and case-crossover studies. Environ Pollut.

[20] Maclure M (1991). The case-crossover design: a method for studying transient effects on the risk of acute events. Am J Epidemiol.

[21] Janes H, Sheppard L, Lumley T (2005). Case-crossover analyses of air pollution exposure data: referent selection strategies and their implications for bias. Epidemiology.

[22] Peng RD, Dominici F, Pastor-Barriuso R, Zeger SL, Samet JM (2005). Seasonal analyses of air pollution and mortality in 100 US cities. Am J Epidemiol.

[23] Gleason JA, Bielory L, Fagliano JA (2014). Associations between ozone, PM2.5, and four pollen types on emergency department pediatric asthma events during the warm season in New Jersey: a case-crossover study. Environ Res.

[24] Strickland MJ, Hao H, Hu X, Chang HH, Darrow LA, Liu Y (2016). Pediatric emergency visits and short-term changes in PM2.5 concentrations in the U.S. state of Georgia. Environ Health Perspect.

[25] Xiao Q, Liu Y, Mulholland JA, Russell AG, Darrow LA, Tolbert PE, Strickland MJ (2016). Pediatric emergency department visits and ambient Air pollution in the U.S. State of Georgia: a case-crossover study. Environ Health.

[26] Darrow LA, Klein M, Flanders WD, Mulholland JA, Tolbert PE, Strickland MJ (2014). Air pollution and acute respiratory infections among children 0-4 years of age: an 18-year time-series study. Am J Epidemiol.

[27] Malig BJ, Green S, Basu R, Broadwin R (2013). Coarse particles and respiratory emergency department visits in California. Am J Epidemiol.

[28] Kim K, Park EY, Lee KH, Park JD, Kim YD, Hong YC (2008). Differential oxidative stress response in young children and the elderly following exposure to PM (2.5). Environ Health Prev Med.

[29] de Oliveira AAF, de Oliveira TF, Dias MF, Medeiros MHG, Di Mascio P, Veras M, Lemos M, Marcourakis T, Saldiva PHN, Loureiro APM (2018). Genotoxic and epigenotoxic effects in mice exposed to concentrated ambient fine particulate matter (PM (2.5)) from São Paulo city, Brazil. Part Fibre Toxicol.

[30] Schulze F, Gao X, Virzonis D, Damiati S, Schneider M, Kodzius R (2017). Air quality effects on human health and approaches for its assessment through microfluidic chips. Genes.

[31] Zhang Y, Salam MT, Berhane K, Eckel SP, Rappaport EB, Linn WS, Habre R, Bastain TM, Gilliland FD (2017). Genetic and epigenetic susceptibility of airway inflammation to PM2.5 in school children: new insights from quantile regression. Environ Health.

[32] Eum KD, Kazemiparkouhi F, Wang B, Manjourides J, Pun V, Pavlu V, Suh H (2019). Long-term NO_2_ exposures and cause-specific mortality in American older adults. Environ Int.

[33] Liu Y, Chen X, Huang S, Tian L, Lu YA, Mei Y, Ren M, Li N, Liu L, Xiang H (2015). Association between air pollutants and cardiovascular disease mortality in Wuhan, China. Int J Environ Res Public Health.

[34] Collart P, Dubourg D, Levêque A, Sierra NB, Coppieters Y (2018). Short-term effects of nitrogen dioxide on hospital admissions for cardiovascular disease in Wallonia. Belgium Int J Cardiol.

[35] Cheng MF, Tsai SS, Wu TN, Chen PS, Yang CY (2007). Air pollution and hospital admissions for pneumonia in a tropical city: Kaohsiung. Taiwan J Toxicol Environ Health A.

[36] Frampton MW, Boscia J, Roberts NJ (2002). Nitrogen dioxide exposure: effects on airway and blood cells. Am J Phys Lung Cell Mol Phys.

[37] Negrisoli J, Nascimento LFC (2013). Atmospheric pollutants and hospital admissions due to pneumonia in children. Rev Paul Pediatr.

[38] Szyszkowicz M, Kousha T, Castner J, Dales R (2018). Air pollution and emergency department visits for respiratory diseases: a multi-city case crossover study. Environ Res.

[39] Dong H, Yu Y, Yao S, Lu Y, Chen Z, Li G, Yao Y, Yao X, Wang SL, Zhang Z (2018). Acute effects of air pollution on ischaemic stroke onset and deaths: a time-series study in Changzhou. China BMJ Open.

[40] Ueda K, Yamagami M, Ikemori F, Hisatsune K, Nitta H (2016). Associations between fine particulate matter components and daily mortality in Nagoya. Japan J Epidemiol.

[41] Zeb B, Alam K, Sorooshian A, Blaschke T, Ahmad I, Shahid I (2018). On the morphology and composition of particulate matter in an urban environment. Aerosol Air Qual Res.

[42] Sarnat JA, Koutrakis P, Suh HH (2000). Assessing the relationship between personal particulate and gaseous exposures of senior citizens living in Baltimore. MD J Air Waste Manag Assoc.

[43] Huang CH, Lin HC, Tsai CD, Huang HK, Lian IB, Chang CC (2017). The interaction effects of meteorological factors and air pollution on the development of acute coronary syndrome. Sci Rep.

